# How honeybees respond to heat stress from the individual to colony level

**DOI:** 10.1098/rsif.2023.0290

**Published:** 2023-10-18

**Authors:** Jitesh Jhawar, Jacob D. Davidson, Anja Weidenmüller, Benjamin Wild, David M. Dormagen, Tim Landgraf, Iain D. Couzin, Michael L. Smith

**Affiliations:** ^1^ Department of Collective Behaviour, Max Planck Institute of Animal Behavior, 78464 Konstanz, Germany; ^2^ Department of Biology, University of Konstanz, 78464 Konstanz, Germany; ^3^ Centre for the Advanced Study of Collective Behaviour, University of Konstanz, 78464 Konstanz, Germany; ^4^ School of Arts and Sciences, Ahmedabad University, 380009, Ahmedabad, Gujarat, India; ^5^ Department of Mathematics and Computer Science, Freie Universität Berlin, 14195 Berlin, Germany; ^6^ Department of Biological Sciences, Auburn University, 36849 Auburn AL, USA

**Keywords:** collective behaviour, superorganism, colony reorganization

## Abstract

A honey bee colony functions as an integrated collective, with individuals coordinating their behaviour to adapt and respond to unexpected disturbances. Nest homeostasis is critical for colony function; when ambient temperatures increase, individuals switch to thermoregulatory roles to cool the nest, such as fanning and water collection. While prior work has focused on bees engaged in specific behaviours, less is known about how responses are coordinated at the colony level, and how previous tasks predict behavioural changes during a heat stress. Using BeesBook automated tracking, we follow thousands of individuals during an experimentally induced heat stress, and analyse their behavioural changes from the individual to colony level. We show that heat stress causes an overall increase in activity levels and a spatial reorganization of bees away from the brood area. Using a generalized framework to analyse individual behaviour, we find that individuals differ in their response to heat stress, which depends on their prior behaviour and correlates with age. Examining the correlation of behavioural metrics over time suggests that heat stress perturbation does not have a long-lasting effect on an individual’s future behaviour. These results demonstrate how thousands of individuals within a colony change their behaviour to achieve a coordinated response to an environmental disturbance.

## 1. Introduction

Collective systems coordinate their behaviour to enable properties that are beyond the limits of any single individual [1]—from flocks of birds evading a predator [2], to army ant trails that self-assemble [3], to epithelial cells sealing wounds [4]. In social insects, individual workers perform different tasks that contribute to colony function, creating a superorganism: a cooperative unit to propagate their genes [5–7]. Workers in these colonies can be organized according to age, experience, genes, physiology, social interactions or some combination [8–15]. There is a long history of research into task allocation in the social insects (reviewed in [16,17]), but recent methodological advances have enabled investigators to track thousands of individuals throughout their entire lives [18–23]. These automated-tracking tools have been employed on colonies in a stable state [18,24–28] or in response to the introduction of foreign stressors such as pesticides or disease [29,30]. However, fewer studies have examined how colony organization adapts to environmental disturbances; previous research in this area has relied on limited or manual observations to examine subpopulations or changes in the behaviours of specific individuals [31,32].

Social insects have a range of preferred temperatures, and a colony can adapt its behaviour to counteract changes in ambient temperature to maintain nest homeostasis [33,34]. The ability of social insects to regulate and maintain a stable nest temperature is considered a major selection pressure for social evolution [34] because temperature fluctuations have a negative impact on brood development, which reduces colony fitness [35,36]. As a result, social insects have developed different adaptations for maintaining a steady temperature profile. In termites, the mound structure allows effective air circulation, thus facilitating both gas exchange and heat loss during bouts of extreme heat [37]. In ants, workers transport brood to different nest regions to develop at suitable temperatures, choosing locations to account for seasonal and even daily variations in nest temperature [38]. Honeybees, however, rear their brood in hexagonal cells where the brood cannot be relocated at will [39,40]. Therefore, honeybees use a suite of behavioural responses to actively modify nest temperature to maintain homeostasis.

Honeybee brood is carefully maintained at 34.5 ± 1.5°C [41]. When ambient temperatures drop, workers vibrate their thoracic muscles to generate heat and cluster tightly together to conserve heat. When ambient temperatures rise, workers use a diverse set of behaviours to cool the nest: fanning air to increase air circulation, collecting and spreading water for evaporative cooling, heat shielding to dissipate heat and even evacuating the nest [32,42–46]. As long as the colony has access to water, workers can maintain nest homeostasis even when ambient temperatures reach as high as 60°C [42]. Although a colony must forage for water to cool their nest when temperature increases, this does not affect the colony’s rate of nectar intake [44]. A previous study on colony and individual responses to heat stress found task switching to be essential for coping with heat stress, though the role of inactive workers, or ‘reserve labour’, was unclear [46]. Overall, these results suggest that colonies adapt to environmental stressors in a way that does not entirely disrupt other colony processes. Furthermore, while the different behavioural responses to heat stress are well recognized, it is unknown how colony-scale reorganization takes place during a heat stress; for example, the roles and distributions of task specialists, generalists and reserved labour remain unclear. At an individual level, it is not known how a bee’s previous role predicts their response to heat stress, and whether there are distinct behavioural groupings structured by age or experience across the entire colony.

In this study, we use automated tracking of individually tagged honeybees to examine how a colony responds to heat stress at both the individual level and the colony level. We experimentally induced an ambient heat stress by heating the room in which the colony was housed. Using continuous tracking data of several thousand honeybees, we examine how the in-nest placement and movement characteristics of bees in the colony change in response to the heat stress. After analysing the colony-wide behavioural changes, we use a generalized analysis framework based on behavioural metrics to label and describe changes in the behaviour of individual bees, and to ask how previous behaviour relates to subsequent behavioural changes during the heat stress. These results describe how individual bees respond to a heat stress, and how individual behavioural changes are organized across thousands of bees in the colony.

## 2. Methods

### 2.1. Overview

**Figure 1.**
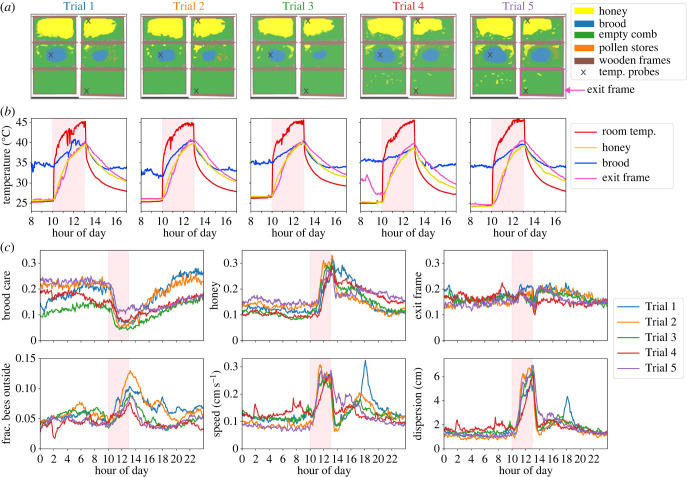
Nest contents, temperature and colony-level response to heat stress. (*a*) Nest contents across all five experimental trials, showing both sides of the three-frame observation hive. The nest entrance is located at the lower right on the exit frame. The X symbols show the locations of the thermocouples for temperature measurements inside the nest. (*b*) Temperature readings in the observation hive room and inside the nest during the heat stress trials. The heat stress was conducted between 10.00 and 13.00 (pink-shaded area). (*c*) Colony-level averages before, during and after the heat stress for all five trials (line colours denote trial and pink-shaded area denotes the heat stress). The top row shows the average fraction of time individual bees spent on brood, honey and the exit frame. The bottom row shows the fraction of the total tracked bees estimated as outside of the hive, as well as the average speed and dispersion of tracked bees. See also electronic supplementary material, figure S1 for analogous plots of average metrics on control days.

The goal of this experiment was to observe how a collective system responds to a heat stress at both the individual level and the colony level. Using an observation hive stocked with individually marked honeybees (see below), we heat stressed the colony between 10.00 and 13.00 on five non-consecutive dates in 2019 (Trial 1: 08–23, Trial 2: 08–25, Trial 3: 08–31, Trial 4: 09–06, Trial 5: 09–09). The day prior to each heat stress trial was used as a control to measure and compare individual- and colony-level behaviour, with the exception of Trial 3, where other manipulations were performed on this day; we therefore omit this day in control versus heat stress comparisons. The heat stress was induced by raising the temperature of the observation hive room from approximately 25°C to approximately 45°C using a personal heater (Bomann Keramik-Heizlüfter HL 1097 CB). Temperature was logged every 10 s using K-type thermocouples (Omega Engineering), which were placed in the observation hive room, and embedded into the nest (honey frame, brood frame and exit frame; figure 1*a*). Thermocouples were connected to ARDUINO UNO 3 boards equipped with a four-channel thermocouple interface (CN0391-ARDZ shield, Analog Devices) running with a custom script to log the temperature of four thermocouples into a csv-file once per minute.

### 2.2. Observation hive and data processing

This study was conducted at the University of Konstanz, Germany (47.6894° N, 9.1869° E) using colonies of the western honeybee *Apis mellifera carnica*. On 10 May 2019, we installed a honeybee colony (4000 unmarked workers and a single queen) into a three-frame observation hive (observation hive dimensions: 490 × 742 mm; ‘Deutsche-Normal’ frames: 395 × 225 mm). From 14 May to 23 September 2019, every 4–5 days, we individually marked 250–400 newborn workers with BeesBook tags [20,21] and introduced them to the observation hive (as in [28]). To create maps of the nest contents (as in [28,47]), we manually outlined the following nest regions onto plastic sheets which were then digitized: honey storage, pollen storage, brood nest, empty comb, wooden frames, peripheral galleries and dance floor (defined by where waggle dances were observed).

The observation hive was recorded at 3 frames s^−1^ from 5 June to 23 October 2019 using 4 Basler acA4112-20um cameras fitted with Kowa LM25XC lenses. The entire experimental apparatus (observation hive, cameras, lighting, temperature loggers, heaters and structural support) was kept in the dark to mimic the lighting conditions of a natural nest; to capture images, we used infrared light (850 nm 3 W LEDs), which the bees cannot perceive [48]. By using the BeesBook tracking system [20,21], we processed the raw images from the video recordings to detect and decode each individually marked bee in the observation hive. For each bee, we obtain tag ID, ID detection confidence, XY position in the nest, bee orientation and time of detection. All data were stored in a PostgreSQL database. To determine the death date for each individual, we used a Bayesian changepoint model (as in [15]).

### 2.3. Behavioural metrics, principal component analysis and clustering

As in our previous work [28], we used behavioural metrics calculated over a specific time bin to quantify the behaviour of each bee throughout the heat stress. These metrics represent space use within the nest (time spent on honey, brood or exit frame; distance from nest exit), detection (time observed, time outside and number of outside trips) and movement/spatial localization (speed, dispersion and fraction of nest visited). See [28] for further details on how these metrics are calculated from trajectory data. The metrics included here are the same as in [28], but with two differences due to the experimental manipulations. Firstly, instead of using ‘time spent on the dance floor’ as a space-use metric, we instead used ‘time spent on the exit frame’, which does not assume that dances are occurring. Therefore, we also omit ‘number of dance floor visits’. Secondly, we do not include ‘speed circadian coefficient’ because this metric is defined using data over an entire day, and here, we compare data for specific periods within the same day (e.g. before, during and after the heat stress). After filtering the raw trajectory data (detection confidence threshold greater than or equal to 0.8; minimum of 10 detections within the time bin), we calculated behavioural metrics averaged over time bins of different sizes, including 1 min, 5 min and 1 h.

A bee’s barcode is not always detected when it is in the observation hive, for example if the bee is upside-down or in a dense crowd of other bees. Therefore, we used both detection and exit distance to estimate when a bee was outside [28]. This procedure uses 1 min binned values of time observed and median exit distance to estimate when a bee exited and subsequently returned to the nest; this time scale is fine enough to resolve when bees enter/exit but long enough to obtain reliable detections when a bee is in the nest. A bee is estimated to have exited the nest in a time bin *t*_exit_ if the time observed in *t*_exit_ is less than a threshold of *t*_obs_ = 2 s, and if the median exit distance in time bin *t*_exit_ − 1 is less than a threshold *d*_exit_ = 18.75 cm (1500 pixels). The bee is considered to have re-entered in bin *t*_enter_ if the time observed in *t*_enter_ is greater than or equal to *t*_obs_. The threshold values used here for *t*_obs_ and *d*_exit_ are the same as used in our previous work [28]. After applying this procedure to estimate whether each bee was inside or outside of the nest for each 1 min time bin, we averaged the results and saved in the 5 min and 1 h binned data structures.

We used data averaged in 5 min bins to plot trends over time (figure 1), and data averaged in 1 h bins to compare behaviour before, during and after the heat stress in subsequent figures. Including all heat stress and control days, we included data from a total of 2974 unique tracked bees.

To perform principal component analysis (PCA), we constructed a data matrix *A*_*ij*_, where each row *i* represents one behavioural hour (i.e. the behavioural metrics for a single tracked bee during a single hour), and the columns *j* = 1, …, 10 are the different metrics (e.g. time spent on brood). *A*_*ij*_ includes data for the hour of the morning period (9.00–10.00 am), i.e. before the heat stress, and the hour of the midday period (12.00–13.00), i.e. during the heat stress, for all heat stress experimental days. Note that it is possible that a bee is still alive but not detected during an entire hour, so the total number of behavioural hours with data for the morning and the midday periods were slightly different. However, this rarely occurred (3.3% of bees were observed during one period and not the other), so we simply include all data for the PCA and clustering steps.

Following standard procedures, we normalized the data matrix **A** so that the column mean is zero and the column standard deviation is 1. We then performed PCA on the resulting matrix to obtain the components.

We use clustering to create a simplified description that captures the primary behaviour differences among individual bees during the morning and heat stress periods. Each cluster represents bees with similar behaviour during the associated time period. We perform Ward hierarchical clustering using the package scipy.cluster.hierarchy in Python. To examine changes in behaviour from before to during the heat stress, clustering is performed separately on behavioural hours from the morning (before the heat stress) and the midday (during the heat stress). This is done by selecting data, i.e. *A*_*ij*_(*hour* = 9.00−10.00) to obtain the morning hour clusters M* and *A*_*ij*_(*hour* = 12.00−13.00) to obtain the heat stress clusters H*. The full dendrogram structure for clustering applied to each period is shown in electronic supplementary material, figure S2. To facilitate analysis along the main axes of variation, five clusters are used for the morning period and four clusters for the heat stress period.

To calculate the per cent difference of cluster assignments from morning to heat stress, we formed a matrix *F*_*ij*_ where each entry is the fraction of bees in the morning cluster *i* that were also in the heat stress cluster *j* (electronic supplementary material, table S1 shows the counts of bees from the morning to heat stress clusters). To generate a null expectation for this overlap between clusters, we calculated $F_{ij}^{*}=m_{i}h_{\;j}$, where *m*_*i*_ is the fraction of observed bees in cluster *i* in the morning and *h*_*j*_ is the fraction of observed bees in cluster *j* during the heat stress. The per cent difference is then calculated as $(F_{ij}-F_{ij}^{*})/F_{ij}^{*}$.

### 2.4. Correlation of behaviour over time

To determine how long the behavioural changes associated with the heat stress lasted, we computed changes in the correlation coefficient for behavioural metrics of individual bees over time. To calculate the correlation across all behaviour metrics (rather than just for individual metrics), we use a vector generalization of the Pearson correlation coefficient. Consider an hour *h*_1_ with corresponding data *X*_*ij*_, and an hour *h*_2_ with corresponding data *Y*_*ij*_, where the index *i* represents an individual bee and *j* each behavioural metric. Before calculating the correlation coefficient, each behavioural metric *j* in both *X*_*ij*_ and *Y*_*ij*_ is first normalized using common factors; for this, we use the per-metric mean and standard deviation of the matrix *A*_*ij*_. The correlation coefficient *C*_*i*_ for bee *i* in hours *h*_1_ and *h*_2_ is then calculated as follows:

2.1 $$C_{i}(h_{1},h_{2})=\frac{\sum_{\;j}X_{ij}Y_{ij}}{\sqrt{\left(\sum_{\;j}X_{ij}X_{ij}\right)\left(\sum_{\;j}Y_{ij}Y_{ij}\right)}}.$$

This correlation is calculated for bees *i* that have data in both *X*_*ij*_ and *Y*_*ij*_. We used *h*_1_ = 9.00–10.00 for both control days and treatment days, and then *h*_2_ in hour increments until 9.00 the following day. The correlation coefficient *C*_*i*_(*h*_1_, *h*_2_) varies between 1 and −1; positive values represent a positive correlation, zero is no correlation and negative values are a negative correlation. This calculation allows us to determine how quickly and for how long the behaviour of a colony deviates during a heat stress, relative to the behaviour on days without a heat stress.

## 3. Results

Following [15,28,49], we introduced barcode-tagged bees into an observation hive and tracked their behaviour continuously over an entire summer. Between 23 August and 9 September 2019, we conducted five heat stress trials. During this time of year, the colony’s nest structure contained honey in the upper frames, brood care areas in the middle frames and empty comb in the lower frames (figure 1*a*). On each experimentally induced heat stress day, room heaters were switched on at 10.00 and off at 13.00. This led to a quick increase in the ambient room temperature, which was followed by an increase in nest temperature (figure 1*b*). Note that in the absence of a heat stress, there are temperature differences among nest areas: while honey and exit frame areas are similar to ambient temperature, brood is maintained at a higher, consistent temperature of approximately 35°C (see time before the heat stress in figure 1*b*). During the heat stress, the temperature on all nest areas—including honey, brood and the exit frame—increased to a maximum of approximately 40°C. However, the brood area temperature increased at a slower rate compared with the honey and exit frame. Given that the brood area temperature was higher than other areas before the heat stress, the overall change was comparatively less during the heat stress than other nest regions.

### 3.1. Colony-level behavioural changes

We observed overall changes in nest use and movement characteristics among bees in the colony during the heat stress. Workers spent less time on central brood areas and more time on honey areas at the top of the nest, and as temperature increased, the number of bees that exited the hive increased (figure 1*c*). In addition, we saw large changes in movement characteristics during the heat stress: on average, bees moved faster and were less localized (figure 1*c*). The latter is demonstrated by the increase in dispersion, which quantifies a bee’s range regardless of its nest location [28]. Although response trends were similar each day, more bees exited the nest during Trial 2 in comparison with other trials, and in the late afternoon after the heat stress during Trial 1, there was an additional short time period with an increase in speed and dispersion. These colony-level patterns demonstrate that bees responded to our experimental manipulations by altering both where and how they move within the nest, and that similar changes were seen during each heat stress trial (figure 1).

### 3.2. Distributions of individual movement and space use

**Figure 2.**
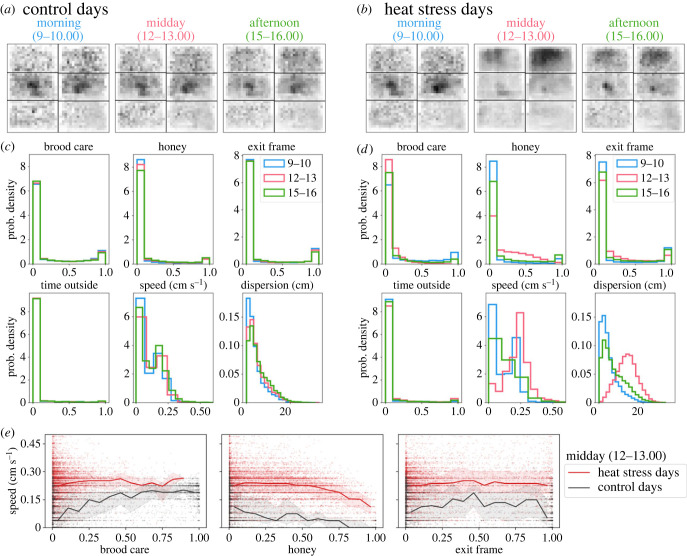
Spatial positioning and movement during heat stress. (a,*b*) The distribution of bees in the nest, showing histograms for hour-long periods in the morning (9.00–10.00), midday (12.00–13.00) and afternoon (15.00–16.00), grouping together (*a*) control days (days prior to heat stress trials) and (*b*) heat stress days. See figure 1*a* for nest contents. (*c*,*d*) Distributions of individual space use and movement metrics for morning, midday and afternoon time periods, showing (*c*) control days and (*d*) heat stress days. (*e*) Conditional distributions of speed as a function of time spent on brood, honey and exit frame, using data for the midday time period (i.e. during the heat stress on heat stress days). The lines show the median, and shaded area the upper/lower quartiles for control and heat stress days. Points show the values for individual bees.

We next examine in further detail how bees responded to the heat stress using the distributions of metrics for hour-long periods at different times of the day—morning (9.00), midday (12.00) and afternoon (15.00)—for both heat stress and control days. Control days include days prior to the heat stress days (see §2), and on these days, we see only small changes in overall space use (figure 2*a*) and the distributions of behavioural metrics at different times of day (figures 2*c*; electronic supplementary material, S1). During the heat stress, figure 1*c* shows that there were large changes in the average space use and movement characteristics among bees in the colony. However, a change in average values can be caused by either a small number of individuals making large changes or a large number of individuals making smaller changes. The large shift in the distributions of the movement metrics of speed and dispersion demonstrates that the latter is true; nearly all bees changed their behaviour during the heat stress (figure 2*d*).

We also ask where the queen went during the heat stress. Although limited to a single individual, the detected trajectory points for the queen before, during and after the heat stress period are shown in electronic supplementary material, figure S6. Similar to the overall trends we observed for workers, during the heat stress, the queen became more active and moved away from the brood area, without exiting the nest (electronic supplementary material, figures S6 and S7).

On control days and in the morning time prior to the heat stress, the distributions of time on brood, honey and the exit frame have a ‘U-shape’, with the highest representation of values for time fractions near 0 or 1 (note, however, that there are many more values near 0 than 1). We use the terminology ‘specialists’ to refer to bees who spend nearly all their time on a specific nest substrate (i.e. time fractions near 1 for a certain substrate). Note that as in our previous work [28], this definition is based on spatial location in the nest and not on observations of performing particular tasks. For example, brood specialists spend nearly all of their time on the brood area. By using this definition, we see that while the average time on honey areas increased during the heat stress, the number of brood and honey specialists decreased during the heat stress. Note that because we use ‘specialist’ to refer to bees that spend a large fraction of time on a particular substrate, the decrease in the number of specialists during the heat stress is linked to the increase in dispersion; if bees are ranging around more of the nest (i.e. higher dispersion values), then they are less likely to remain on a single nest area, and this is exactly what we observe during the heat stress (i.e. fewer specialists).

While figure 1*c* shows that on average, more bees exit the hive during the heat stress, figure 2*d* shows that this increase is accounted for by some bees spending more time outside, while the majority of bees remained inside the nest during heat stress. By 15.00 in the afternoon, i.e. after the heat stress, we see a decrease in dispersion and a re-emergence of both brood and honey specialists, but that the distributions of all behavioural metrics have not yet returned to their pre-heat stress levels.

Given the large shift in the distribution of individual speeds during the heat stress (figure 2*d*), we further examine the relationship between speed and substrate occupancy in the nest. By using the midday hour, figure 2*e* compares the distribution of speed conditional on the fraction of time spent on either brood, honey or the exit frame for control versus heat stress days. On control days, bees spending a high amount of time on brood tend to be more active (i.e. higher average speed over time) than other individuals working inside the nest (figure 2*e*). Previous work saw a similar trend, where a greater proportion of time spent on brood was associated with higher median speed values (see fig. 4D and cluster 5 in [28]). During the heat stress, however, there is no longer a clear association between substrate occupancy and movement speed. This reinforces the general conclusion that, during heat stress, all bees move faster and range over a larger area and are less likely to spend all their time on a specific substrate.

### 3.3. Generalized behavioural responses to heat stress using principal component analysis and clustering

To build upon the previous sections, we next take a generalized approach that combines all behavioural metrics using PCA and clustering, to investigate how individuals change their behaviour during the heat stress [28,50]. PCA extracts the dominant axes of behavioural variation, i.e. the relative weightings of the behavioural metrics that explain the largest percentage of variance in the data, and reduces data by projecting onto these dominant (orthogonal) axes. Positive/negative PCA weightings indicate greater/lesser values of a metric relative to an average that includes both morning and heat stress hours (see §2).

**Figure 3.**
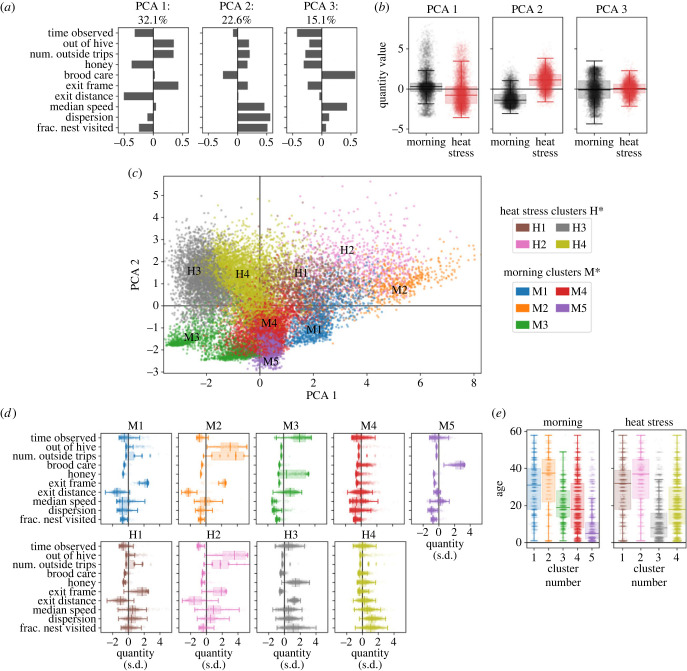
PCA and clustering describe dominant behavioural differences before and during heat stress. PCA is performed on the behavioural metrics for bees in the morning (pre-heat stress, 9.00–10.00), and during the heat stress (12.00–13.00), using data for all heat stress trials. (*a*) The first three PCA components, showing the weightings of each and the fraction of variance explained. (*b*) Distributions of PCA embeddings during the morning (black) and heat stress (red). (*c*,*d*) Ward hierarchical clustering is applied to both the morning period and the heat stress period (see also electronic supplementary material, figure S2). We use five clusters to describe dominant behavioural differences for the morning hour labelled M1–M5, and four clusters for the heat stress hour labelled H1–H4. (*c*) The embeddings along the first two PCA axes and (*d*) distributions of the behavioural metrics for each cluster. (*e*) Age distributions of bees in each cluster. See also electronic supplementary material, figure S3 for the PCA embedding with points coloured by age.

The first three PCA components explain 69.8% of the variance across all metrics, with the first two components accounting for 54.7%. Figure 3*a* shows the weightings of the metrics in first three PCA components. PCA 1 represents location in the nest, with positive projections representing bees close to the exit and/or going outside. PCA 2 represents differences in activity levels, with positive projections associated with higher speed, dispersion and fraction of nest visited. PCA 3 represents specific in-nest space use, and positive projections are strongly weighted by increased time spent on brood areas. Beyond these, the fraction of variance explained by the subsequent components is [10, 6.4, 4.7, 3.7, 1.9, 1.4, $1.8\times 10^{-28}\%$]. Note that the heuristic 'Kaiser rule' suggests that the first three PCA components are significant to retain, with the fourth component as marginal since its variance explained is equal to that of a single input variable [51]; we therefore plot only the first three components. We see differences in the distributions of the first three PCA components during the heat stress, but most noticeably in PCA 2, where the increase of the average projection represents the general increase in activity levels (see also figures 1 and 2). We also see a decrease in the median projection onto PCA 1, which reflects the general shift of bees spending time on honey areas, far from the nest exit, during the heat stress.

We use clustering to form a simplified description that captures the main differences in behaviour among individual bees during the morning and heat stress periods. Clustering is applied separately to the morning and heat stress data (i.e. assignments are based on bee behaviour during the respective time periods), and we use a four-cluster division for the morning and a four-cluster division for the heat stress (electronic supplementary material, figure S2). This allows us to identify links between morning and heat stress behaviours by mapping which bees belonged to which clusters in the morning and during the heat stress. Note that, as in our prior work [28], the clustering approach is used as a descriptive tool to describe the space of continuous variation in individual behaviour.

During the morning period (M), the five-cluster division defines the following behavioural groups: (M1) near the exit, (M2) going outside, (M3) on honey stores and far from the nest exit, (M4) other nest regions and peripheral areas and (M5) mainly on brood (figure 3*c*,*d*). During the heat stress period (H), the four-cluster division defines: (H1) near the exit, (H2) going outside, (H3) mostly on honey stores and (H4) high dispersion (figure 3*c*,*d*). Note that because the M* and H* clusters are defined independently, bees from any of the morning clusters can be subsequently observed in any of the heat stress clusters (e.g. bees in M1 can be in any of the clusters H1 through H4).

**Figure 4.**
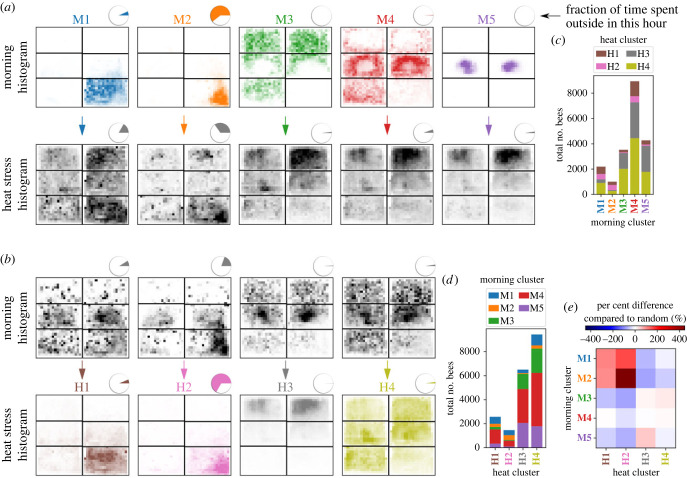
Nest location for each behavioural cluster from the morning to the heat stress periods. (*a*,*b*) Nest location in the morning (9.00–10.00, top) and during the heat stress (12.00–13.00, bottom), conditional on the behavioural cluster of each bee on the heat stress day. Pie chart insets show fraction of time spent outside for bees in each cluster. See also electronic supplementary material, figure S4, which overlays the histograms for the behavioural clusters. (*a*) Morning behavioural clusters (M1–M5), and (*b*) heat stress behavioural clusters (H1–H4). (*c*) Stacked bar graph displaying the number of bees in each heat stress cluster, as a function of their morning cluster membership. (*d*) Same as (*c*), but for the number of bees in each morning cluster as a function of heat stress cluster. See also electronic supplementary material, figure S5 for analogous per-trial stacked bar graphs. (*e*) Per cent difference of cluster assignments from morning to heat stress, comparing actual values with random assignments. See also electronic supplementary material, table S1 for the counts of bees that transitioned from each morning to heat cluster.

We use the cluster representation to map behaviour between the morning and the heat stress period, and identify how an individual’s earlier behaviour affects their role during the heat stress. Bees that were near the exit (M1) or already going outside (M2) tended to have similar space use during the heat stress (i.e. a tendency to belong to heat stress clusters H1 or H2; figure 4). Bees in these clusters (M1 and M2; H1 and H2) tended to be older than bees in other clusters (figure 3*e*). Bees that were on brood areas in the morning (M3) moved to the top of the nest during the heat stress (figure 4*a*), with a tendency to belong to heat stress cluster H3 (on honey areas) (figure 4*e*). The clusters (M5 and H3) tended to include the youngest bees (figure 3*e*). Bees in the other morning clusters (M3 and M4) tended to move towards honey areas at the top of the nest (figure 4*a*), but with weaker predictability for which heat stress cluster they would join. M4 bees, for example, were nearly equally likely to be in any of the four heat stress clusters (figure 4*e*).

We can also use the four heat stress behavioural clusters (H1–H4) to examine the details of where these individuals were before the heat stress (figure 4*b*). Bees that were near the exit during heat stress (H1) or went outside (H2) tended to be closer to the exit during the morning period than other bees; comparing these two clusters, however, H2 bees were the closest to the exit and spent the most time outside in the morning period. Bees mostly on honey stores during the heat stress (H3) tended to be on brood areas during the morning period. Bees in H3 as well as those that had high dispersion during the heat stress (H4) tended to spend little time on the exit frame in the morning period.

In the behavioural clusters, we see differences in the median ages of bees. In the morning period, M5 (brood workers) tends to include younger bees, M3 and M4 tend to include middle-aged bees and M1 and M2 have the highest median age. During the heat stress, median ages representing young, middle-aged and old bees are represented by clusters H3, H4, and H1 and H2, respectively. Looking at the PCA, we see that PCA 1 is clearly linked to age (electronic supplementary material, figure S3). This demonstrates that there is overlap in the ages of bees between the morning and heat stress behavioural clusters and age differences are indeed linked to behavioural differences both before and during the heat stress.

To summarize, we see that the bees that evacuate during a heat stress tend to be older and were already present close to the nest exit or outside of the nest before the heat stress (M1 and M2 → H1 and H2). In addition, we see that bees that were engaged in brood care in the morning (M5) move towards the top of the nest, thereby clearing out the brood nest, but not evacuating the nest entirely.

### 3.4. Colony recovery after the heat stress

To determine how long the behavioural changes associated with the heat stress lasted, we computed the correlation coefficient for behavioural metrics of individual bees between a starting hour *h*_1_ and a later hour *h*_2_. We set *h*_1_ to be the morning hour (9.00–10.00) of a particular day, and increase *h*_2_ for successive hour periods until the next day (see §2). We use this correlation coefficient to compare how behaviour changes over time on control days versus heat stress days.

**Figure 5.**
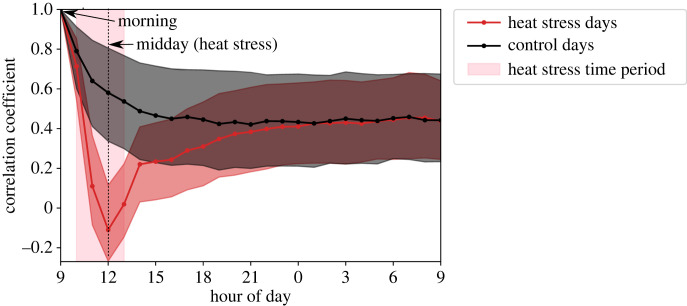
Behavioural correlation over time. Correlation coefficient as a function of time, calculated as the correlation of a bee’s behavioural metrics in the morning (9.00) with each subsequent hour. Control days (days before heat stress) are shown in black; heat stress days are shown in red. Pink shading denotes the heat stress time period. Lines show the median of correlation across all bees, and shaded area shows the upper/lower quartiles.

Even without a heat stress, bees change their behaviour over time, and therefore, we see a decrease in the behavioural correlation coefficient on control days (black line, figure 5). On heat stress days, however, the correlation coefficient decreases rapidly, with median values becoming negative at hour 12.00 (red line, figure 5). Once the heat stress ends, the correlation increases as the bees ‘recover’ from the heat stress and resume their typical behavioural repertoires. By the evening on the heat stress days (approx. 21.00), the correlation coefficient is no different than on control days (figure 5). This suggests that while bees do change their behaviour dramatically during the heat stress, the perturbation does not have a long-lasting effect on their future behavioural trajectories.

## 4. Discussion

In this study, we used automated tracking to examine how a colony coordinates its response to heat stress at both the individual level and colony level. At the colony level, the heat stress induced bees to move away from brood areas and towards honey areas at the top of the nest, as well as some bees to exit the nest. Nearly all bees increased their average activity levels during the heat stress. The increase in activity and individual dispersion led to a temporary loss of ‘specialists’ (used here to refer to individuals who spend nearly all of their time on a specific nest substrate) during the heat stress [28]. By using PCA and clustering, we described the dominant behavioural groups and used these groups to link individual behaviour before and during the heat stress. This revealed that individual bees engaged in brood care in the morning moved towards the top of the nest during the heat stress and that the bees that evacuated the nest during the heat stress tended to be older and were already spending time close to the nest exit or outside of the nest before the heat stress. Following the heat stress, the overall trends of bee substrate occupancy and activity levels soon began to return to their pre-heat stress levels, suggesting that their behavioural states or tendencies are retained (i.e. are not completely modified due to the heat stress). We quantified this using the correlation coefficient of all behavioural metrics over time, which shows that while bees do change their behaviour dramatically during the heat stress, the perturbation does not have a long-lasting effect on the future behaviour of individuals.

During a heat stress, bees take on different activities such as water collection, fanning, heat dissipation and nest evacuation. Typically, fanning happens near the exit to increase airflow into and out of the nest [43,52]. Because of their proximity to the exit, it is possible that H1 cluster bees participated in fanning. Bees in the H4 cluster had the highest dispersion values; among other activities, these bees may have been active in receiving and dispersing water [32,44]. As in [31,32], we found that the number of bees outside of the nest increased during the heat stress. Although we did not attempt to distinguish between pollen, nectar and water foragers, the increase in bees outside of the nest is consistent with pollen and nectar foragers continuing their work alongside a set of water foragers during the heat stress [44,53].

Prior research suggests that ‘task switching’ functions as a primary driver of bees adopting thermoregulation-related tasks, though the role of recruitment of reserved labour is not clear [31,54]. In [31], the proportion of bees standing still inside the nest was used as a proxy for inactivity. Johnson found that during the heat stress, the proportion of inactive bees did not change, and in subsequent days, the inactive bees were not consistently inactive—leaving their role ambiguous. In our experiments, however, we observed that the average activity of nearly all bees increased during the heat stress, which suggests a general colony-level response and a potential role for inactive or ‘reserved’ individuals in mitigating heat stress. These differences could potentially be due to the higher temperatures reached in our experiments or simply due to the timescale of our analysis—we examined behaviour averaged over a (relatively) long period using continuous tracking, instead of an instantaneous distribution of specific metrics among bees at a given time.

Automated barcode tracking enables the long-term identification and tracking of individual bees. While we used trajectory data to calculate behavioural metrics, a promising direction for future research is to combine barcode tracking with supervised machine learning techniques to automatically detect specific behavioural events [22,25]. Combining these methods would facilitate not only the analysis of individual behavioural changes over extended time periods but also the identification of specific behaviours such as fanning or standing still. These additional data could be used, for example, to examine switching dynamics between behaviours, or how fanning behaviour spreads among bees as temperature increases in the nest [55].

At the colony level, a sufficient number of bees must be assigned to each task to ensure an adequate overall response that maintains safe nest temperatures for the developing brood. One proposed mechanism for achieving this is the ‘frequent quitting/task switching’ model, in which bees frequently quit their current task and seek out another [54]. Simulations demonstrated that this simple mechanism can effectively allocate bees to tasks that require more attention, if the bees are attracted to these tasks by stimuli [54]. To describe the response of different individuals, previous studies with social insects have employed a threshold model in which individuals in a group differ in their response threshold levels [16,49,56,57]. The effective individual threshold for a bee to engage in fanning behaviour has been shown to depend on social context (i.e. the number of other bees present) as well as on the rate of temperature increase [58,59]. In addition, it was seen that fanning activity spreads across individuals, and bees previously engaged as fanners were more likely to initiate the spread of fanning to other surrounding bees [55]. This emphasizes that not only external stimuli but also interactions with other bees contribute to an individual’s decision of what to do next [15].

Future research could use targeted manipulations to investigate how the age distribution and within-nest structure affect the general distribution of response behaviours among bees in the colony. For example, removing all or a proportion of older or younger bees, systematically, and observing how the distribution of individual responses changes highlight such differences between individual and collective action. While the observation hive had honey on the top frames and brood in the middle frames during our trials, manipulations could systematically vary these placements to see how this influences the overall response. Although we observed bees move onto honey areas, repeating the experiment after swapping the honey frames to the lower part of the nest could answer whether the redistribution of bees is specific to honey areas or if it is driven by other thermoregulatory factors that favour movement to comb areas far from the exit, regardless of what they contain. Furthermore, while we investigated behaviour by using age-matched cohorts, future research could evaluate or change individual aspects other than age that are related to a bee’s heat response, such as gene expression [60] or neuromodulatory factors [61] or previous tasks performed and thermoregulatory experience. In summary, by using data from thousands of individually marked bees, we showed how a colony’s organization changes and adapts to rapidly changing environmental condition within their nest.

## Data Availability

All data associated with this study are freely available online through Zenodo [62], with data for the queen provided in a separate repository [63]. Code to reproduce the results in this paper is available from GitHub digital repository: https://github.com/jacobdavidson/beeṡheatstresṡ2019data. All data are available from Zenodo digital repository: doi.org/10.5281/zenodo.7298798. Additional figures and information are provided in electronic supplementary material [64].
